# The role of perfectionism in body dissatisfaction

**DOI:** 10.1186/2050-2974-1-2

**Published:** 2013-01-22

**Authors:** Tracey D Wade, Marika Tiggemann

**Affiliations:** 1 School of Psychology, Flinders University, GPO Box 2100, Adelaide 5001 SA, Australia

## Abstract

**Background:**

Body dissatisfaction is a robust risk factor for disordered eating and is thought to be especially problematic in the presence of high levels of perfectionism. The aim of the current study was to investigate what types of perfectionism were associated with body dissatisfaction. Participants were 1083 women aged 28 to 40 years, with a mean age of 35 years (SD=2.11). Self-reports on perfectionism (using the Frost Multidimensional Perfectionism Scale), weight, height, desired weight, and current and ideal figural stimuli were analysed for the current study. Two measures of body dissatisfaction were utilised: discrepancy between the current and desired weight, and discrepancy between the current and ideal figural stimuli.

**Results:**

Linear regressions controlling for current body mass index (BMI)/current silhouette examined the relationship between desired BMI/silhouette and simultaneous entry of the 6 subscales of the perfectionism measure. A lower desired BMI was associated with higher levels of Concern over Mistakes and Organisation, and a smaller ideal silhouette was associated with higher levels of Concern over Mistakes and Doubt about Actions and Organisation.

**Conclusions:**

These findings confirm the pertinence of different dimensions of perfectionism to body dissatisfaction, and suggest avenues to explore in terms of universal prevention work.

## Background

Perfectionism has come to be viewed as an important maintaining factor of disordered eating. In the transdiagnostic theory of eating disorders, Fairburn, Cooper and Shafran [1] assert that clinical perfectionism is one of four core mechanisms that maintain eating disorder pathology and if it were to be ameliorated then “…a potent additional network of maintaining mechanisms would be removed thereby facilitating change” (p. 516). In the cognitive-interpersonal model of anorexia nervosa [2], perfectionism/cognitive rigidity is one of the four postulated maintaining factors. In addition, the three-factor theory by Bardone-Cone and colleagues [3] implicates the interaction between high perfectionism, high body dissatisfaction, and low self-esteem in the growth of bulimic behaviour. In support of these theoretical positions, research consistently shows perfectionism to be elevated in people with eating disorders and people recovering from eating disorders compared to controls [4,5].

However the precise nature of the construct of perfectionism continues to be debated in the literature. Perfectionism has been proposed to be a multidimensional construct by two groups of theorists. The first construct, proposed by Hewitt and Flett [6], focuses on the interpersonal components of perfectionism, and the associated 45-item scale is divided into three subscales. The *selforiented* perfectionism subscale relates to setting high standards for achievement and self-criticism for not meeting standards. The *otheroriented* perfectionism subscale includes items that relate to having high standards for other people that are unrealistic. The *sociallyprescribed* perfectionism subscale items are related to perceiving that other people hold unrealistically high standards for the individual.

The second theory proposes a 6 factor construct for perfectionism, measured using the Frost Multidimensional Perfectionism Scale (FMPS [7]): Personal Standards (setting high standards), Concern over Mistakes (negative reactions to mistakes and perceiving mistakes as failures), Doubts about Actions (doubting one’s own performance), Parental Expectations (parents setting high standards), Parental Criticism (parents criticising for mistakes), and Organisation (organisation and neatness). Factor analyses have consistently shown a two factor solution, consisting of adaptive (achievement striving) perfectionism (Personal Standards and Organisation), and maladaptive evaluative concerns (Concern over Mistakes, Doubt about Action, Parental Expectations, and Parental Criticism) [8,9]. Achievement striving is typically associated with healthy functioning while maladaptive evaluative concerns is more consistently associated with psychopathology [8]. There is one exception to this general finding, which is that elevated levels of both types of perfectionism are associated with eating disorders [10]. Thus it has been suggested that elevated levels of *both* types of perfectionism confers most risk for disordered eating [11].

Less studied is the way in which (if at all) perfectionism relates to the risk factors for eating disorders. This is particularly true for body dissatisfaction which is considered to be a robust risk factor for eating pathology [12] along with a related construct, weight concern. These constructs have been found to predict the development of disordered eating in adolescent samples across a number of studies, resulting in their status as “the best confirmed and most potent risk factor” (page 131) for eating disorders [13]. While one study has shown that body image concerns (as measured with the Body Attitudes Test [14]) was positively associated with both adaptive and maladaptive perfectionism [15], tests of the three-factor theory show that adaptive and maladaptive perfectionism interacted with body dissatisfaction to predict binge eating, while only adaptive perfectionism interacted with body dissatisfaction to predict self-induced vomiting [3]. An examination of the associations between perfectionism and body appreciation showed a significant positive association with organisation and a significant negative association with maladaptive evaluative concerns [16].

Thus further research is required in order to better understand the complexities of which type of perfectionism relates to body dissatisfaction. This will inform us as to whether perfectionism only relates to the more extreme ends of the spectrum (e.g., eating disorders) or also to more normative states such as body dissatisfaction. Such knowledge can benefit universal prevention work where the audience is young people who are not identified as at risk for eating disorders and the aim is to decrease recognised risk factors for disordered eating and thereby provide “immunisation” for eating disorders. Therefore the aim of the present research was to examine the relationships between different dimensions of perfectionism and two indicators of body dissatisfaction, the discrepancy between current and desired body mass index and the discrepancy between current and ideal figural silhouettes. We hypothesised, in line with previous suggestions [11], that body dissatisfaction would be associated with both adaptive and maladaptive evaluative concerns perfectionism.

## Methods

### Participants

Participants were 1083 women aged 28 to 40 years, with a mean age of 35 years (SD=2.11). They were participants in the third wave of data collection in a study of female twins. The sample of twins approached to participate in this study was originally derived from a cohort registered as children with the Australian Twin Registry (ATR) during 1980–1982, in response to media appeals and systematic appeals through schools and has previously been described in a number of publications e.g. [17]. Female-female twins (n=2320 twins or 1140 complete twin pairs) who had participated in at least one of two waves of data collection, Wave 1 during 1989–1992 when the twins were aged 18–25 years, and Wave 2 during 1996–2000 when the median age of the sample was 30 years, were approached during 2001–2003 to participate in a third wave of data collection. Of these, 1,083 individual twins actively consented to participate (47%). Participation at Wave 3 was not predicted by the number of eating problems at Wave 1. The total protocol at Wave 3 consisted of two parts, a telephone interview and a self-report questionnaire. The Flinders University Clinical Research Ethics Committee approved the data collection process and written informed consent was obtained after the procedures had been fully explained.

### Measures

Self-report measures relating to perfectionism utilized the FMPS [7]. This included the following subscales: Personal Standards (7 items e.g., “I have extremely high goals”), Concern over Mistakes (9 items e.g., “If I fail partly it is as bad as being a complete failure”), Doubt about Actions (4 items e.g., “I usually have doubts about the simple everyday things I do), Parental Expectations (5 items e.g., “My parents set very high standards for me”), Parental Criticism (4 items e.g., “My parents never tried to understand my mistakes”), and Organisation (6 items e.g., “Organisation is very important to me”). Higher scores indicated higher levels of perfectionism on all the sub-scales. The subscales demonstrated high internal consistency in the current study, with all alphas ≥ .82.

The figural stimuli developed by Stunkard, Sorenson and Schlusinger [18] were used to assess current and ideal figures. Following Fallon and Rozin [19], participants were first asked to rate adult female figures using the question “Which silhouette is closest to what you look like now?” (Current) and then they were asked to rate the figures again answering “Which silhouette would you prefer to look like now?” (Ideal). The difference between these values has often been interpreted as an indication of body dissatisfaction e.g. [20].

In addition, a telephone interview was conducted consisting of the Eating Disorder Examination (EDE) 14th edition [21], part of which assessed self-reported weight and height, desired weight, and highest adult body weight. The discrepancy between desired and current body mass index (BMI) was used as a second measure of body dissatisfaction. Both the discrepancy measures were significantly correlated with the weight and shape concern measure from the EDE, .50 and .48 respectively with the Current-Ideal discrepancy and .51 and .46 with the BMI discrepancy.

### Statistical analyses

Given the purpose was to examine relative strengths of associations between perfectionism and desired BMI and ideal figure, the twin data were not adjusted for correlated observations but examined using linear regressions. In the first analysis, desired BMI was included as the dependent variable, and current BMI was entered as a covariate at the first step, followed by all 6 subscales of the FMPS at the second step. In the second analysis, the ideal silhouette was included as the dependent variable, and current silhouette was entered as a covariate at the first step, followed by all 6 subscales of the FMPS at the second step. We used a covariate approach rather than using the discrepancy measures as outcome variables as the latter leads to a loss of reliability.

## Results

### Descriptives

The means and standard deviations are shown in Table 1 and the correlations between all variables are shown in Table 2. It can be seen that the Concern over Mistakes subscale correlated with the other sub-scales in the FMPS ranging from .27-.38 and, in line with previous findings [7], Organisation did not correlate significantly with any of the other subscales. The difference between current and desired BMI correlated strongly with the discrepancy between current and ideal figure ratings (.70). In addition, current figure correlated strongly with self-reported BMI (.81).

**Table 1:** Descriptive statistics for the sample

**Variable**	**N**	**Mean** (**SD**)	**Range**
Age	1002	34.97 (2.11)	28.10-39.97
Body mass index	998	24.09 (4.91)	14.20-63.98
Desired body mass index	978	21.93 (2.57)	14.20-33.06
Current silhouette	1013	4.42 (1.20)	1-9
Ideal silhouette	1013	3.55 (0.66)	1-7

**Table 2:** Bivariate correlations between the variables

**Variable**	(**2**)	(**3**)	(**4**)	(**5**)	(**6**)	(**7**)	(**8**)
Current – desired BMI (1)	.70**	.20**	.003	.20**	−0.002	.15**	.08*
Ideal – Current Silhouette (2)		.17**	.02	.17**	-.03	.17**	.08*
Concern over mistakes (3)			.35**	.27**	.34**	.28**	.38**
Personal standards (4)				.27**	-.02	.35**	.21**
Doubt about actions (5)					-.02	.35**	.21**
Organisation (6)						.03	.05
Parental criticism (7)							.62**
Parental expectations (8)							

** p≤0.001; * p<0.05.

### The relationship between perfectionism and body dissatisfaction

The zero-order correlations in Table 2 also indicate that both discrepancy measures correlated positively with Concern over mistakes, Doubt about actions, Parental criticism and Parental expectations, when considered individually. The aim of the linear regression analysis, however, was to assess the unique contribution of each facet when considered together. As the linear regressions controlled for current size (BMI or silhouette), the analyses addressed the question of the extent to which perfectionism predicted individuals’ ideals, over and above the effect of their current size. In addition, the results can be interpreted as informing our understanding of factors that contribute to body dissatisfaction. The results of these regressions are shown in Table 3.

**Table 3:** **Linear regressions with the Frost subscales as independent variables simultaneously**, **controlling for current weight or size**

**Variable**	**Desired body mass index**	**Ideal silhouette**
	**Beta** (**p**) **Effect Size**	**Beta** (**p**) **Effect Size**
Concern over mistakes	−0.07 (0.005) 0.19	−0.15 (<0.001) 0.29
Personal standards	0.01 (0.68) 0.03	0.04 (0.23) 0.08
Doubt about actions	−0.03 (0.25) 0.08	−0.08 (0.02) 0.15
Organisation	−0.05 (0.01) 0.17	−0.12 (<0.001) 0.27
Parental criticism	−0.03 (0.20) 0.09	0.02 (0.63) 0.03
Parental expectations	0.02 (0.33) 0.07	-.002 (0.95) 0.004

Note: Effect size = Cohen’s *d* where >0.2 is small, >0.5 is medium and >0.8 is large.

Two measures of perfectionism had significant and independent associations with desired BMI when controlling for current BMI, namely Concern over Mistakes (maladaptive perfectionism) and Organisation (adaptive perfectionism), indicating a higher level of perfectionism was associated with a lower desired BMI. At the first step, where Current BMI was entered, the R^2 ^change was .68, and the addition of the perfectionism subscales was associated with R^2 ^change of .01, such that 69% of the total variance was accounted for by all the entered independent variables (adjusted R^2^). In terms of effect size contribution, the largest contribution was made by Concern over Mistakes, followed by Organisation.

Three measures of perfectionism had significant and independent associations with the Ideal silhouette when controlling for the Current silhouette, namely Concern over Mistakes and Doubt about Actions (maladaptive forms of perfectionism) and Organisation (adaptive perfectionism), indicating a higher level of perfectionism was associated with a thinner Ideal. At the first step, where Current figure was entered, the R^2 ^change was .26, and the addition of the perfectionism subscales was associated with R^2 ^change of .05, such that 31% of the total variance was accounted for by all the entered independent variables (adjusted R^2^). In terms of effect size contribution, the largest contribution was made by Concern over Mistakes, followed by Organisation and then Doubt about Actions.

## Discussion

Body dissatisfaction is an important risk factor for disordered eating, and yet is also considered a normative concern in women [22]. The aim of the current research was to examine which specific forms of perfectionism are associated with body dissatisfaction. We hypothesised that both sub-scale measures of adaptive perfectionism and maladaptive concerns perfectionism would be associated with our two discrepancy measures of body dissatisfaction. While individual correlations indicated associations between our discrepancy measures and all four FMPS subscales that assess maladaptive forms of perfectionism, multivariate consideration of the associations with the body discrepancy measures implicated roles for *both* maladaptive and adaptive forms of perfectionism. Desired weight and size were most strongly associated with Concern over Mistakes and Organisation when controlling for current BMI and size respectively. Specifically, a lower desired BMI was associated with higher levels of Concern over Mistakes and Organisation, and a smaller ideal silhouette was associated with higher levels of Concern over Mistakes and Doubt about Actions and Organisation.

Our results differs somewhat from prior research [14] which found significant univariate associations between body attitudes and both maladaptive (i.e., concern over mistakes) and adaptive (i.e., personal standards) forms of perfectionism. In addition, a multivariate examination of variables contributing to body appreciation showed a positive association with organisation [16] i.e., higher levels of organisation were associated with higher levels of body appreciation. This latter study also found a much greater contribution of maladaptive perfectionism to body appreciation than organisation, whereas the current study indicates associations of a similar effect size.

While maladaptive perfectionism has been shown across many different studies to be associated with poor outcomes in psychopathology, including eating disorders [10], there has been far less examination of the relationship between Organisation and disordered eating and its risk factors. Organisation has previously been implicated as part of a temperament that underlies a common familial liability to anorexia nervosa [23] and should be considered for inclusion in further research of body dissatisfaction and disordered eating. Our findings may indicate that organisation does not become unhelpful unless it is in the presence of high levels of maladaptive perfectionism, as suggested by previous research examining eating disorder symptoms [11]. In terms of how perfectionism exerts an effect on body dissatisfaction, it may be that the highest levels of body dissatisfaction which can act as risk factors for the later development of disordered eating are associated with high levels of both organisation and a concern over mistakes that results in criticism of oneself as a person. To the degree that organisation indicates a need to exert control over the environment, those people high on organisational traits may display more extreme efforts to control body appearance. Any resultant failure to meet the desired standards in this domain results in self-criticism and negative affect which is suggested to be part of the vicious cycle that perpetuates unhelpful perfectionism [24].

This research should be interpreted within a number of limitations. First, the research has been conducted with women with a mean age of 35 years and may not be generalizable to adolescents, the demographic at which prevention efforts are typically targeted. Second, while there is no reason to suggest that a twin sample is not representative of a non-twin sample, it may be that having a sibling of the same age increases body comparisons in women. Third, our measures of body dissatisfaction only represent one dimension of this construct, and further work is needed to investigate the relationships with other dimensions of body dissatisfaction and perfectionism, as only a few such studies exist [14-16]. Finally, there is a need to establish a prospective link between perfectionism and body dissatisfaction which may further clarify this relationship.

These findings suggest that perfectionism is pertinent to the normative state of body dissatisfaction. Given the role of body dissatisfaction in increasing risk for disordered eating, this suggests that targeting perfectionism may be of benefit in buffering young people against the development of disordered eating. One piece of research [25] has investigated an intervention that targeted perfectionism in middle adolescence which significantly reduced maladaptive evaluative concerns compared to two other conditions (media literacy informed by inoculation theory, which suggests that building skills to resist social persuasion will prevent the development of health-risk behaviours and a control condition). While there were no overall significant changes in shape and weight concern or dieting, there were a large proportion of people in the high risk participants in the perfectionism group (i.e., having levels of weight and shape concern ≥ 4 at baseline) who experienced clinically significant reductions in both variables (57%). In contrast only 38% and 33% experienced clinically significant reductions for weight and shape concern in the other two conditions respectively, with 25% and 17% experiencing such reductions for dieting. Longitudinal research linking perfectionism and body dissatisfaction is required before further prevention research in this area is conducted.

## Conclusions

These findings confirm the general pertinence of both maladaptive and adaptive perfectionism to body dissatisfaction. Future research should investigate whether decreasing concern over mistakes perfectionism alone might decrease body dissatisfaction, in line with previous findings that suggest decreasing this variable is associated with large reductions in eating disorder psychopathology [26]. In addition, the suggestion that high scores on *both* maladaptive and adaptive forms of perfectionism are problematic for body dissatisfaction should be subject to empirical test.

## Competing interests

The authors declare that they have no competing interests.

## Authors’ contributions

TDW and MT conceived the analyses, TDW did the analyses and TDW and MT drafted the manuscript, and both authors read and approved the final manuscript.
